# A Classic Presentation of Roseola Infantum

**DOI:** 10.7759/cureus.52504

**Published:** 2024-01-18

**Authors:** Micah Pippin, Gloria Laws

**Affiliations:** 1 Family Medicine, Louisiana State University Health Sciences Center, Alexandria, USA

**Keywords:** viral febrile illness, exanthema subitum, sixth disease, rash in children, viral exanthem, roseola infantum

## Abstract

Roseola is a common viral exanthem of childhood, most frequently affecting infants and toddlers before age three. The syndrome is characterized by an abrupt onset of high fever, which, upon resolution, yields to a centrally located maculopapular rash that spreads peripherally. This report describes the case of an 18-month-old child whose fever and defervescence rash provide insight into the typical presentation and progression of this pervasive yet mostly benign condition.

## Introduction

Roseola infantum, also known as exanthema subitum and sixth disease, is a ubiquitous childhood viral syndrome characterized by high fever followed sequentially by a distinctive defervescence rash [1]. The condition is present globally and affects nearly all children before the age of three years [2-3]. The diagnosis is made clinically by identifying the illness' predictable progression, which is routinely self-limited, requiring symptomatic treatment only [1-3]. While a benign disease course is typical of roseola, a strong association with febrile seizures exists, and, in rare cases, significant neurologic sequelae may occur in the immunocompromised [4-5].

Here, we present a classic case of roseola infantum managed expectantly with a complete resolution of all symptoms two days after the initial presentation.

## Case presentation

An 18-month-old male with no past medical problems and an up-to-date immunization history presented to our family medicine clinic with his mother, who provided a history. The mother reported a high fever followed by a spreading rash. The patient was born at term via uncomplicated, normal spontaneous vaginal delivery and exhibited appropriate development. Four days previously, he developed a fever approaching 104 degrees Fahrenheit (40 degrees Celsius), recorded on a home rectal thermometer. The fever was persistent and only temporarily responded to antipyretics, including acetaminophen and ibuprofen. Other than mild rhinorrhea and a non-productive cough, the patient demonstrated no further symptoms. No abdominal discomfort, diarrhea, nausea, or vomiting were observed. His oral intake was unaffected, and he had an unchanged number of wet diapers. His mother observed that he had been somewhat more irritable. No change in consciousness, abnormal movements, or seizure-like activity were reported. On the third day, the patient's fever resolved, and he began to exhibit a diffuse rash. The eruption started on the patient's chest and moved peripherally to the abdomen, upper extremities, neck, and face. The mother reported that the rash did not seem pruritic as the patient had not scratched and did not seem bothered by the outbreak. No other household contacts demonstrated similar symptoms.

The patient's vital signs were documented as a heart rate of 105 beats per minute, a respiratory rate of 26 breaths per minute, blood pressure of 90/55 mmHg, a rectal temperature of 98 degrees Fahrenheit (37 degrees Celsius), and an oxygen saturation of 99% on room air pulse oximetry. His height was recorded at 83 cm (33 inches), and his weight was 12 kg (26 lbs), which placed him at the 60th and 50th percentiles for age, respectively.

**Figure 1:**
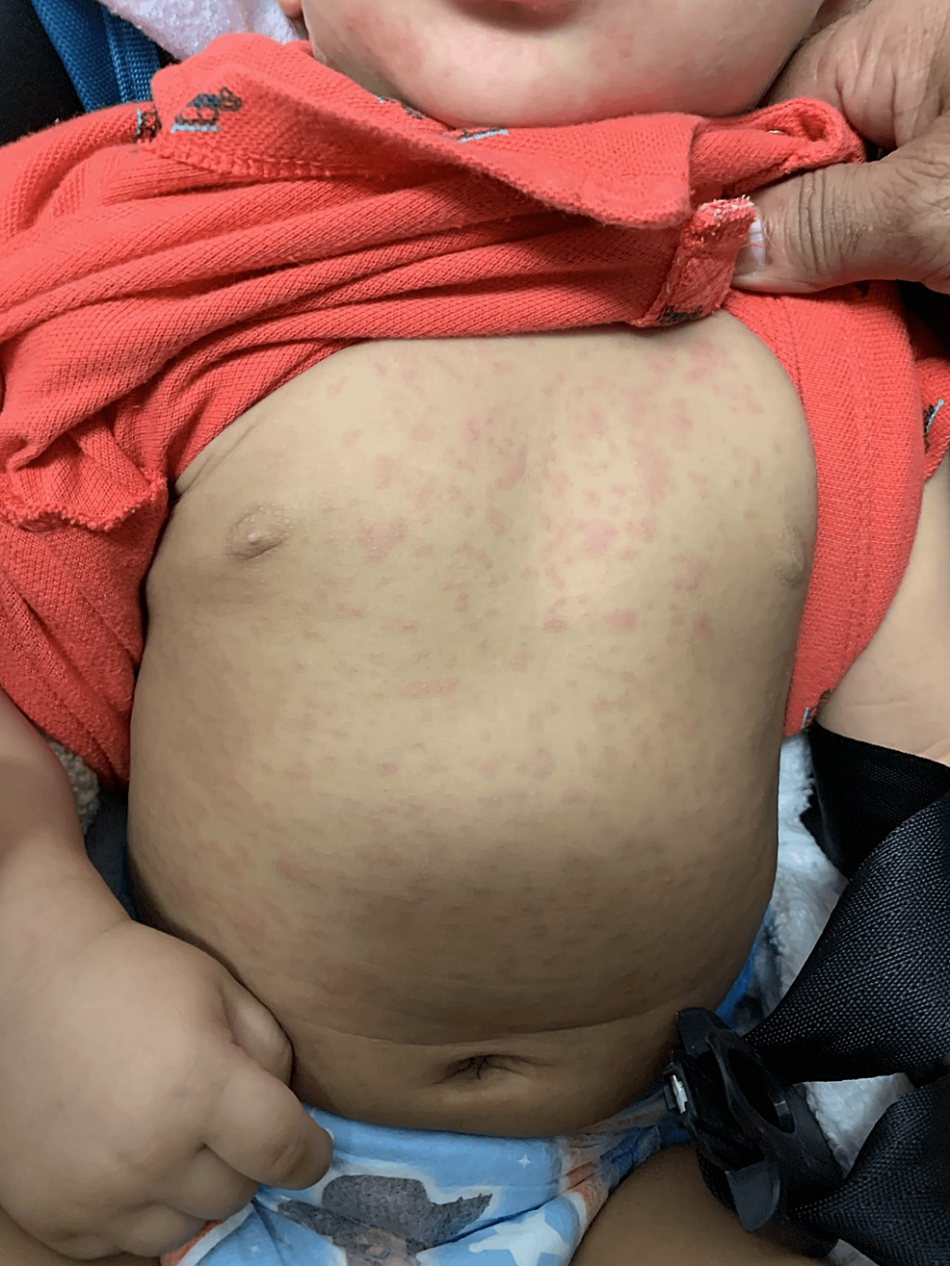
Classic defervescence rash associated with roseola infantum

On physical examination, the patient's skin demonstrated an erythematous, pink, non-scaling, blanching, macular rash centrally distributed on the torso and extending peripherally to the neck, face, and upper extremities (Figure 1).

The child was well-appearing and appropriately playful. The remainder of the physical examination was not notable. Moist mucous membranes, a clear oropharynx, a negative otoscopic investigation of the ear, and absent lymphadenopathy were documented. The lungs were clear to auscultation, and there was no increased breathing work or intercostal retractions. A cardiac examination revealed normal heart sounds with no murmurs, rubs, or gallops. The abdomen was soft and non-tender. Further inspection of the skin revealed an appropriate capillary refill time.

Based on the presentation and typical defervescence rash, the patient was diagnosed clinically with roseola with reassurance provided and expectant management recommended. The patient and his mother returned for a follow-up in one week. They reported a complete resolution of all symptoms two days following their previous visit and six days following the initial symptoms.

## Discussion

Roseola infantum was first described in 1910 and was named for its rose-colored rash [1]. The moniker sixth disease was designated to this syndrome as it was the sixth common childhood rash to be named, and the epithet exanthema subitem was also adopted, which translates to sudden rash [1]. The causative organism is the B variant of human herpesvirus (HHV)-6 and, less frequently, HHV-7, which both belong to the genus Roseolovirus and are members of the Betaherpesviridae subfamily of herpesviruses [1,6]. The viruses have a worldwide prevalence, with most children acquiring infection by two years of age and nearly 100 percent of the global population being seropositive by three years [7]. Ten to 45 percent of febrile illnesses in United States infants have been attributed to HHV-6 [8]. Viral replication occurs abundantly in the salivary glands, resulting in a primarily respiratory droplet transmission mode [3-4]. Following primary infection, the virus remains latent within the host's cells, similar to other herpesviruses [1,4].

The classic presentation of roseola is characterized by one to five days of fever followed by abrupt defervescence and a subsequent rash lasting one to two days [1-4]. The fever is often high and may exceed 104 degrees Fahrenheit (40 degrees Celsius) [8]. The skin eruption typically involves erythematous to rose-pink, 2 mm to 5 mm, blanching, nonpruritic macules and papules with an initial central distribution on the trunk and subsequent peripheral extension to the neck and extremities [8]. Additional history is variable, with some patients asymptomatic and others demonstrating symptoms including cough, congestion, irritability, malaise, and possible diarrhea [2]. Some patients will demonstrate uvulo-palatoglossal erythematous papules known as Nagayama spots [4,8]. 

Rubeola (measles) is another childhood febrile viral illness that presents with a similar appearing exanthem and must be considered in the differential diagnosis [2]. However, the rubeola rash is found initially on the face and expands caudally in contrast to roseola's central outbreak and peripheral advancement [2]. Also, patients with rubeola are generally more ill-appearing, and pathognomonic Koplik spots are found on the buccal mucosa as opposed to roseola's Nagayama papules seen on the uvula and soft palate [2,4,8].

The diagnosis of roseola is clinically directed, and laboratory and radiographic evaluations are generally not indicated [1-4]. The disease course is self-limited, and treatment is supportive with rest, hydration, and antipyretics [1-4,5,8]. The prognosis is favorable, with minimal risk of complications or adverse outcomes [2].

Febrile seizures are the most frequent complications of roseola and have been reported in 10 to 15 percent of cases [5,8]. A significant number of childhood febrile convulsions have been attributed to the viral syndrome, and it is the most likely precipitator of febrile seizures in children under two years of age [4-5,9]. While a history of febrile seizures due to roseola will increase a child's risk of future seizures, the nature of the convulsions is benign [9]. Generally, it does not require any intervention [9].

Although rare, reactivation of latent HHV-6 and HHV-7 viruses in primarily immunocompromised hosts may cause significant pathologic manifestations [10-11]. These patients most commonly experience neurologic complications such as encephalitis, but cases of myocarditis, hepatitis, pneumonitis, and gastrointestinal disease have been reported [1,11-12].

## Conclusions

Roseola is a globally prevalent viral syndrome recognized for its high fever and subsequent defervescence exanthem. Most children will be affected before three years of age, and physicians should consider the condition among their differentials in febrile toddlers. The diagnosis is made clinically, and the course is mainly self-limited, requiring only reassurance and supportive care. Physicians should be aware of the association between roseola and febrile seizures and be ready to educate family members on the benign nature of this frequent complication. While rare, latent virus reactivation in immunocompromised patients may result in significant sequelae, primarily manifesting in neurologic conditions.
